# Crack Monitoring Method for an FRP-Strengthened Steel Structure Based on an Antenna Sensor

**DOI:** 10.3390/s17102394

**Published:** 2017-10-20

**Authors:** Zhiping Liu, Kai Chen, Zongchen Li, Xiaoli Jiang

**Affiliations:** 1 Laboratory of Intelligent Manufacture and Control, Wuhan University of Technology, Wuhan 430063, China; lzp@whut.edu.cn; 2 Faculty of Mechanical, Maritime and Materials Engineering, Delft University of Technology, 2628 Delft CD, The Netherlands; z.li-8@tudelft.nl (Z.L.); X.Jiang@tudelft.nl (X.J.)

**Keywords:** antenna sensor, cracking, FRP-strengthened steel structure, FRP thickness, resonant frequency, sensitivity

## Abstract

Fiber-reinforced polymer (FRP) has been increasingly applied to steel structures for structural strengthening or crack repair, given its high strength-to-weight ratio and high stiffness-to-weight ratio. Cracks in steel structures are the dominant hidden threats to structural safety. However, it is difficult to monitor structural cracks under FRP coverage and there is little related research. In this paper, a crack monitoring method for an FRP-strengthened steel structure deploying a microstrip antenna sensor is presented. A theoretical model of the dual-substrate antenna sensor with FRP is established and the sensitivity of crack monitoring is studied. The effects of the weak conductivity of carbon fiber reinforced polymers (CFRPs) on the performance of crack monitoring are analyzed via contrast experiments. The effects of FRP thickness on the performance of the antenna sensor are studied. The influence of structural strain on crack detection coupling is studied through strain–crack coupling experiments. The results indicate that the antenna sensor can detect cracks in steel structures covered by FRP (including CFRP). FRP thickness affects the antenna sensor’s performance significantly, while the effects of strain can be ignored. The results provide a new approach for crack monitoring of FRP-strengthened steel structures with extensive application prospects.

## 1. Introduction

Fiber-reinforced polymers (FRPs) are widely used in applications including aerospace engineering, bridge fabrication, construction engineering and marine engineering because of their numerous advantages, such as their light weight and high strength, and FRPs have great potential for use in structural manufacturing and reinforcement [1,2,3,4]. Jiang et al. [5] studied an impact detection system based on a fiber Bragg grating (FBG) sensor array and multiple signal classification (MUSIC) algorithm to determine the location and the number of low velocity impacts on a carbon-fiber-reinforced polymer plate. In long-term service, cracks will inevitably appear on steel structures that have been strengthened using FRPs under the influence of external loading and the usage environment [6,7]. Colombi et al. [8] investigated fatigue crack growth in steel beams that were strengthened using carbon-fiber-reinforced polymers. Yu et al. [9] presented a numerical study of the effects of the degree of initial damage, the bond configuration and the crack type on the fatigue behavior of retrofitted specimens using the boundary element method. The accumulated damage in these structures would affect structural safety and could have disastrous consequences. Therefore, effective monitoring of cracks under FRP reinforcement is significant in order to understand the failure mechanisms of FRP-strengthened steel structures and ultimately maintain their safety.

However, the bonding of FRP materials means that it is difficult to detect concealed crack growth due to fatigue loading using common inspection methods. The main difficulty in monitoring cracks in FRP-strengthened steel structures is that the cracks are covered by the anisotropic FRP layer. There are few literature references on crack propagation monitoring of FRP-strengthened steel structures based on eddy current testing (ECT). Li et al. [10] showed that eddy current pulsed thermography (ECPT) could be used to detect impact damage on FRP-strengthened steel structures. Yikuan et al. [11] used Lamb waves to monitor fatigue crack propagation and detect fatigue crack initiation in FRP-strengthened steel plates. Ma et al. [12] found that acoustic emission (AE) techniques were effective in revealing crack processes in FRP-strengthened reinforced concrete (RC) columns. However, these methods have limitations when applied to the quantitative research on crack detection. In addition, the methods are expensive to implement on a large scale because of the associated labor and wiring costs, and they are generally range-limited because of their power requirements. Based on previous investigations of antenna sensors, this paper proposes a new type of dual-substrate antenna sensor to realize passive and wireless monitoring of cracks on FRP-strengthened steel structures.

Referring to studies of crack detection using antenna sensors, Deshmukh et al. [13] first proposed a surface crack detection method for use on metallic surfaces that used a rectangular microstrip patch antenna. Their results indicated that the resonant frequency of the antenna decreased with increasing crack length. The crack detection sensitivity was 29.6 MHz/mm, and the antenna was able to detect crack propagation with sub-millimeter resolution. Mohammad and Huang [14] investigated fatigue crack length measurements using a patch antenna sensor and analyzed the influence of the crack closure effect. Mohammad and Gowda [15] studied the crack orientation detection capabilities of antenna sensors by measuring the resonant frequencies *f*_10_ and *f*_01_ in two directions. In their paper, a rough crack direction identification method was proposed that extended the application range of patch antenna sensors in crack detection. Cook et al. [16] investigated the effects of nonlinear crack shapes on the parallel and perpendicular resonant modes of a patch antenna, and these effects were quantified through simulations and measurements. Yi et al. [17] designed a slotted patch antenna sensor and applied it to fatigue crack detection; the results showed that the resonant frequency decreased with increasing strain. Liu et al. [18] studied the mechanism of crack detection when using a patch antenna sensor based on meander technology, analyzed the effects of cracks on the current distribution, and discussed a method of oblique crack identification based on the relative resonant frequency variation.

In the method of making cracks on specimens, Liu et al. [18] made straight cracks of 0.5 mm width parallel to the length direction of the patch on the central line of the floor. Deshmukh et al. [13] designed and machined a compact tension (CT) specimen according to American Society for Testing and Materials (ASTM) standards (E647-00). A small groove is fabricated at the edge of the floor, and a nearly straight-line crack is produced by fatigue test. Cook et al. [12] made nonlinear wedge cracks at the edge of the floor.

In antenna sensor substrate research, Yi and Wang [19] investigated the strain testing performances of patch sensors with different substrate thicknesses; the strain transmission efficiencies of antenna sensors with substrate thicknesses of 0.79 mm and 1.58 mm were obtained, while the effects of substrate thickness on strain testing linearity and wireless access distance were also analyzed. Yi and Vyas [20] studied the influence of temperature on the relative dielectric constant of a substrate, and the effects of temperature changes in Rogers 6202 and Rogers 5880 materials on the strain test results from their sensors were compared. Wang et al. [21] found that as the edge effect became more obvious with increasing substrate thickness, the resonant frequency could be affected; however, the edge effect of the microstrip antenna sensor could only be neglected if the substrate thickness was much smaller than the width of the patch antenna sensor structure.

In conclusion, fatigue crack monitoring using antenna sensors offers the advantages of passive and wireless measurements. The existing research is mainly aimed at metal materials. The crack detection sensitivity, identification of the crack direction and the effects of the thickness of a single (relatively thin) substrate on test performance have been studied. However, for the crack monitoring of steel plates that have been covered by FRP, there has been little research on the crack monitoring performance of antenna sensors with multilayer substrates and there have also been few studies on the effects of the substrate properties, e.g., weak conductivity and thickness, on crack monitoring performance.

In this paper, a crack monitoring method using a dual-substrate antenna sensor is proposed for crack damage measurement on FRP-strengthened steel structures. The anisotropic FRP layer is regarded as the second external substrate layer of the antenna sensor. In Section 2, we propose a fundamental resonant frequency analytical model for the proposed dual-substrate antenna sensor based on conformal mapping, transmission lines and cavity model methods. The establishment of a suitable model and related settings for numerical studies are described in Section 3. The crack monitoring sensitivity for FRP-strengthened steel structures when using the proposed antenna sensor is presented in Section 4. Section 5 provides an analysis of the effects of the properties of the FRP on crack monitoring performance, and the work includes: (i) use of comparison experiments to analyze the crack detection performance when a carbon fiber reinforced polymer (CFRP) with weak conductivity is regarded as a substrate; (ii) study of the influence of FRP thickness on the crack detection performance of the antenna sensor. In Section 6, strain and crack coupling experiments are used to analyze the effects of structural strain on crack detection performance.

## 2. Crack Monitoring Mechanism of Dual-Substrate Antenna Sensor

**Figure 1:**
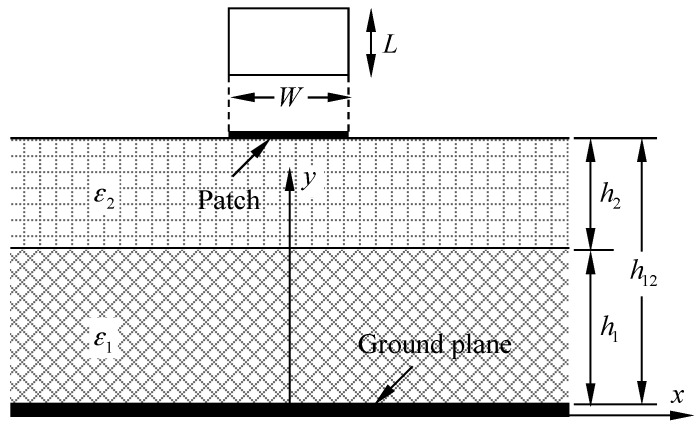
Cross-section of dual-substrate antenna sensor.

To monitor cracking in an FRP-strengthened steel structure, an antenna sensor without a ground plane is arranged on the top surface of the FRP layer, and a dual-substrate antenna sensor model composed of four “steel plate-FRP-substrate-patch” layers is fabricated. Figure 1 shows the cross-section of the dual-substrate antenna sensor, where *L* and *W* are the length and width of the patch, respectively; $\varepsilon_{1}$ and $\varepsilon_{2}$ are the relative dielectric constants of the FRP and the substrate, respectively; *h*_1_ and *h*_2_ denote the thicknesses of the FRP layer and the substrate, respectively; and *h*_12_ is the distance from the ground plane surface to the substrate surface.

Based on a transmission line model, the formula used to calculate the resonant frequency of the dual-substrate antenna sensor is given as

(1) $$f=\frac{c}{2\sqrt{\varepsilon_{r,eff}}}\cdot \frac{1}{L+2\Delta L}$$

where *c* is the speed of light in free space, and the expressions for the effective dielectric constant $\varepsilon_{r,eff}$ and the additional electrical length due to the fringing effect represented by ΔL are respectively presented as

(2) $$\varepsilon_{r,eff}=\frac{\varepsilon_{1}\varepsilon_{2}{\left(q_{1}+q_{2}\right)}^{2}}{\varepsilon_{1}q_{2}+\varepsilon_{2}q_{1}}$$

(3) $$\Delta L=0.412h_{12}\frac{\left(\varepsilon_{r,eff}+0.3\right)\left(W_{e}/h_{12}+0.264\right)}{\left(\varepsilon_{r,eff}-0.258\right)\left(W_{e}/h_{12}+0.813\right)}$$

Here, $q_{1}$ and $q_{2}$ are the effective filling factors. Based on the conformal mapping theory of a microstrip antenna, the effective filling factors are calculated as follows [22]:

(4) $$q_{1}=\frac{h_{1}}{2h_{12}}\left\{1+\frac{\pi }{4}-\frac{h_{12}}{W_{e}}\times \ln\left[\frac{2W_{e}}{h_{1}}\sin\left(\frac{\pi h_{1}}{2h_{12}}\right)+\cos\left(\frac{\pi h_{1}}{2h_{12}}\right)\right]\right\}$$

(5) $$q_{2}=1-q_{1}-\frac{h_{12}}{2W_{e}}\ln\left(\frac{\pi W_{e}}{h_{12}}-1\right)$$

The expression used by Wheeler [23] to evaluate the effective line width $W_{e}$ is given as:

(6) $$W_{e}=W+\frac{2h_{12}}{\pi }\ln\left[17.08\left(\frac{W}{2h_{12}}+0.92\right)\right]$$

Where, the effective filling factor $q_{1}$ and $q_{2}$ is introduced to determine the fundamental resonant frequency of the crack in an FRP-strengthened steel structure that is detected using the dual-substrate antenna sensor. The effective line width $W_{e}$ can be obtained based on a combination of the total thickness of the dual-substrate antenna and the patch width. The effective filling factor can then be calculated using the thicknesses of the dielectric layers, including the FRP and the substrate, along with the effective line width. The effective dielectric constant is characterized using the relative dielectric constant and the effective filling factor for each dielectric layer, and thus the additional electrical length is affected. This paper presents a method based on antenna sensors for crack monitoring in FRP-strengthened steel structures. By inputting the relative dielectric constants and thicknesses of the dielectric layers, such as the FRP and the substrate, the fundamental resonant frequency of the dual-substrate antenna sensor can then be obtained.

When a crack appears or propagates on the ground plane, the current on the ground plane surface will be forced to flow around the crack tip, which will increase the antenna current path and thus reduce the resonant frequency. A crack that is perpendicular to the length of the patch only disturbs the current path of the TM_10_ mode and reduces the resonant frequency *f*_10_, and has no influence on the TM_01_ mode and *f*_01_. Therefore, the crack length can be identified quantitatively based on the resonant frequency offset.

## 3. Numerical Investigation

**Figure 2:**
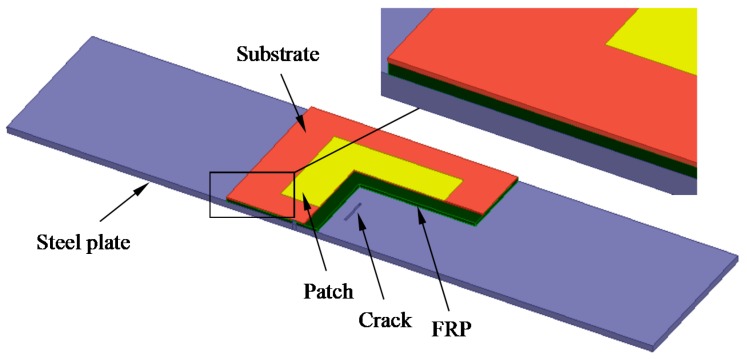
Multi-sectional view of the dual-substrate antenna sensor.

The antenna design was simulated using the commercial electromagnetic simulation software HFSS^TM^, and the crack monitoring model of the dual-substrate antenna is shown in Figure 2. An antenna sensor without a ground plane is arranged on the top surface of the FRP to form a dual-substrate antenna sensor with four “steel plate-FRP-substrate-patch” layers.

A Q235 steel plate was selected as the ground plane material. The patch was made from copper and the substrate was made from FR-4, while a glass fiber reinforced polymer (GFRP) with excellent mechanical properties was also chosen. The geometrical dimensions and the material properties of each layer are shown in Table 1.

In the numerical model, the distance between the radiation air cavity and the antenna sensor is more than one fourth of the wavelength of the unsolved resonant frequency. A second-order cardinal number is chosen as the basis function for the finite element algorithm, and the mesh iteration time and the convergence accuracy are set.

**Figure 3:**
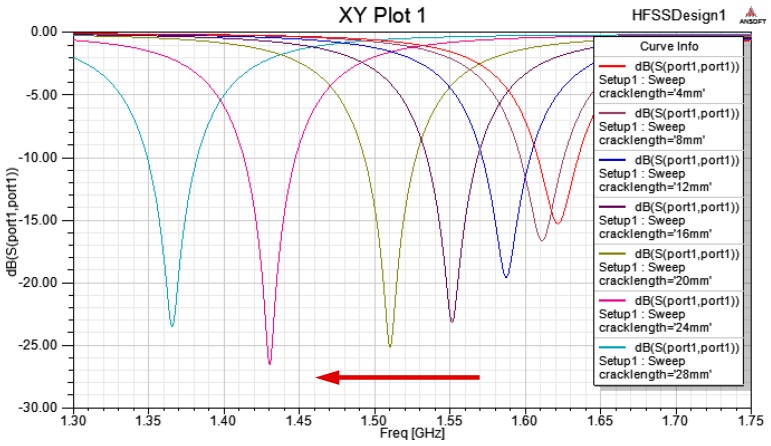
Effects of crack length on the S_11_ parameters of the patch antenna.

The echo loss curve S_11_ is extracted, and the resonant frequency of the dual-substrate antenna sensor is obtained. The effects of the crack length on the S_11_ parameters of the patch antenna are shown in Figure 3.

## 4. Sensitivity Analysis of Antenna Sensors for Crack Monitoring

### 4.1. Sensitivity Analysis

**Figure 4:**
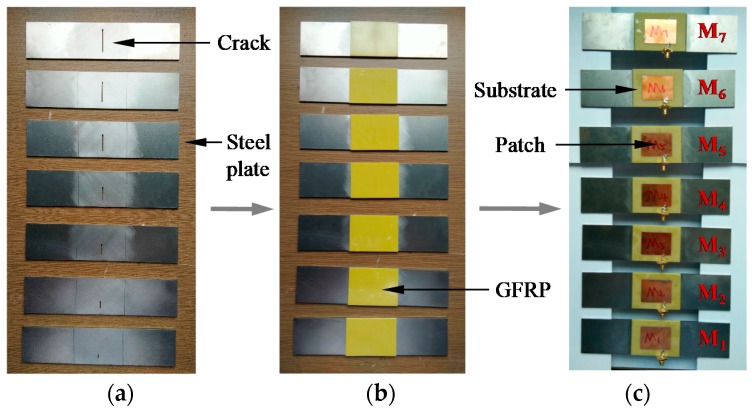
Specimen fabrication process stages: (**a**) steel plates with various crack lengths; (**b**) cracked steel plates when strengthened using glass fiber reinforced polymer (GFRP); (**c**) finished specimens.

An antenna sensor with a two-layer “substrate–patch” structure without a ground plane is designed to validate the simulation results. The substrate and the patch are composed of FR-4 composite (supplied by Huayi Plastic Materials, Shenzhen, China) and copper, respectively, and the two are integrated using etching techniques. Figure 4 shows a sample production process, including (a) seven steel plates with crack lengths that differ with a step size of 4 mm; (b) cracked steel plates when strengthened by GFRP layers; (c) use of epoxy resin AB glue (supplied by Shenneng Adhesives Wholesale, Jinhua, China) to bond the antenna sensor on the GFRP surface and constitute a series of specimens denoted by M_1_–M_7_, with crack lengths of 4 mm, 8 mm, 12 mm, 16 mm, 20 mm, 24 mm, and 28 mm, respectively. A through crack with 0.5 mm width was cut by EDM (electrical discharge machining) for specimens M_1_ to M_7_ with a 0.002 mm machining accuracy.

**Figure 5:**
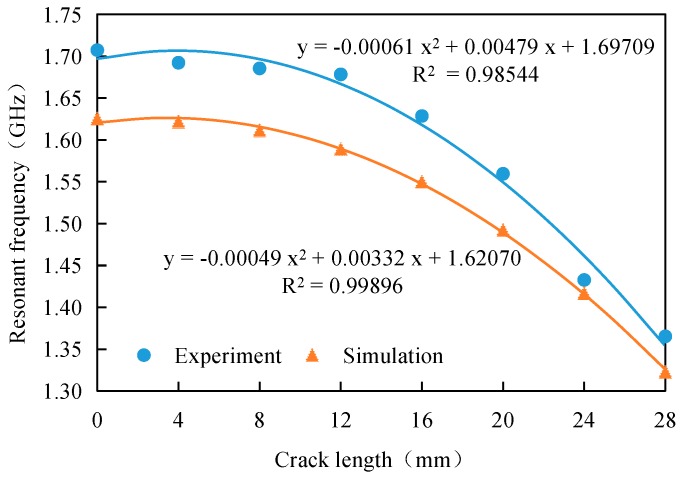
Relationship between resonant frequency and crack length.

A vector network analyzer (VNA) is used to measure the resonant frequency of the antenna sensor, and the results are compared with those of the simulations, as shown in Figure 5. Every data point in Figure 5 is an average of three measurements.

The figure shows that the experimental results show good agreement with the simulation results. When the crack length increases, the resonant frequency of the antenna sensor then decreases, but the resonant frequency offset increases with increasing crack length. When the crack length is small (i.e., less than the width of the patch by one third), the resonant frequency offset is not obvious; however, when the crack is longer, the resonant frequency offset increases significantly. With increasing crack length, the slope of the resonant frequency vs. crack length curve gradually increases, while the sensitivity of the antenna sensor also increases. The curves for the experimental data and the simulation data are both fitted in the figure above. The results show that the resonant frequency of the antenna sensor has a parabolic relationship with the crack length, and this is identical to the conclusion given in the literature [24].

In Figure 5, there is a discrepancy between experiment results and simulation results. The resonant frequencies obtained by the experiments are larger than those obtained by simulation. The reason for this might be the mismachining tolerance of the stability of the Sub-Miniature-A (SMA) joint, the thickness of the glue, the manufacturing tolerance of the antenna sensor and cracks.

Table 2 gives the sensitivity values for the antenna sensor that were obtained from the resonant frequency vs. crack length relationship curve based on Figure 5.

As shown above, with increasing crack length, the sensitivity of the antenna sensor gradually increases, and this indicates that the antenna sensor is more sensitive for the identification of long cracks. When the crack length is 28 mm, the sensitivity values from the simulations and the tests are 29.37 MHz/mm and 24.12 MHz/mm, respectively. This shows that cracks in a steel structure with GFRP reinforcement can be detected using the antenna sensor with the sensitivity reported in [9]. The sensitivity errors in the numerical and experimental studies mainly originate in the antenna sensor manufacturing process, the welding performance of the feeders, and the crack machining accuracy. After a preliminary calibration process, the effects of these errors on the actual crack detection process are fairly small.

### 4.2. The Sensitivity of Small Scale Crack Length

**Figure 6:**
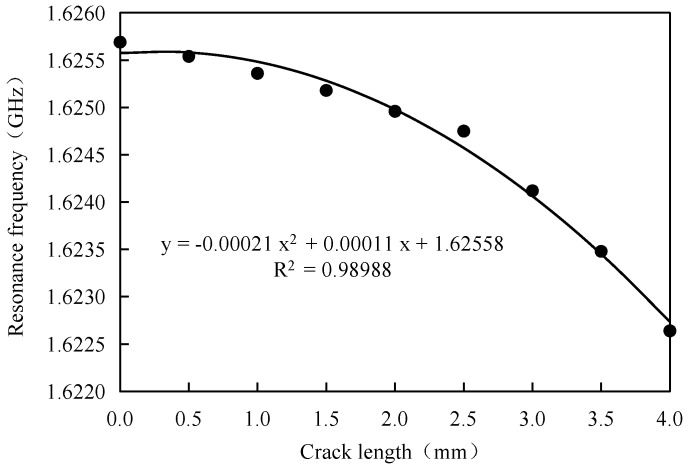
The offset of resonant frequency under small scale variation.

In order to further discuss the sensitivity of small scale crack length, a numerical simulation is adopted. The geometrical dimensions of the antenna sensors and material properties are set as the same with those in Table 1; the crack step length is 0.5 mm and the crack length variation range is from 0 mm to 4 mm. Figure 6 represents the offset of resonant frequency under small scale variation.

Figure 6 indicated that the offsetting value under a variation value with 0.5 mm crack step length is larger than 0.15 MHz. As the accuracy of the Vector network analyzer can be 0.01 MHz, the resonant frequency’s offsetting value of 0.15 MHz can be exactly identified, which indicates that the minimum crack variation value and minimum crack length are both smaller than 0.5 mm.

Based on the resonant frequency–crack length fitted equation represented in Figure 6, the antenna sensor’s sensitivity of small scale crack size can be obtained, as shown in Table 3.

It can be learned from this table that the sensitivity increases with the increasing of crack length, which agrees with the conclusion from Section 4.1. In Section 4.1, the sensitivity of a longer crack is significantly higher than that of a shorter crack, nevertheless, it still can be identified accurately.

### 4.3. The Influence of Crack Shape on Crack Detection

**Figure 7:**
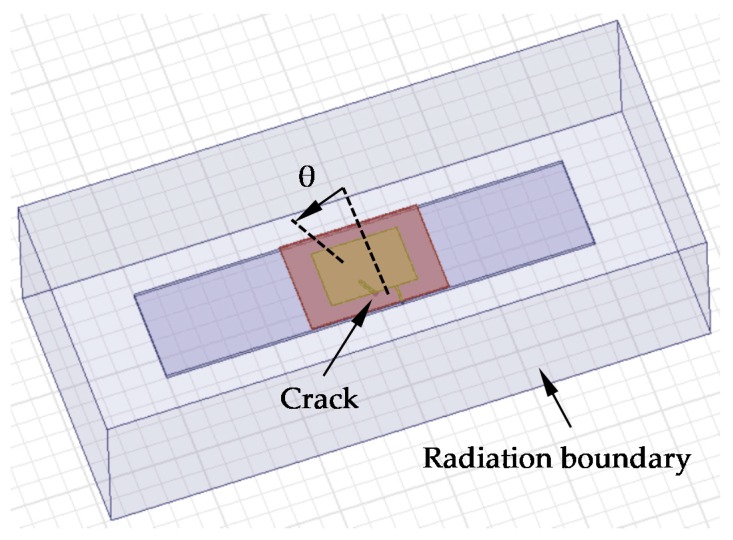
Simulation model for crack orientation.

It is worth mentioning that the crack shape (including crack orientation) has an influence on crack detection. In order to discuss the detectability and accuracy, the influence of crack orientation was investigated. Figure 7 shows numerical models with different crack directions. In Figure 7, the model parameters are all equal to those in Table 1; the crack length is 4 mm and the step size of θ, which represents the crack angle, is 15° in this set of numerical simulations. The resonant frequency of different crack directions are extracted from the S_11_ curve, as shown in Table 4.

In order to identify crack direction, the identification index *R* can be defined by the following formula

(7) $$R=\frac{f_{01}}{f_{10}}$$

Table 4 shows the *R* value of cracks with different directions.

Table 4 indicates that when the crack angle increases, the resonant frequency *f*_10_ increases, while the resonant frequency *f*_01_ decreases, which indicates that the identification method proposed in this paper is still applicable for oblique cracks, and that the crack shape has no influence on the ability of the detection method proposed in this paper. Table 4 shows that the identification index *R* decreases with the increase of crack angle, which means the crack direction can be estimated by the value of *R*, therefore detection accuracy can be improved.

**Figure 8:**
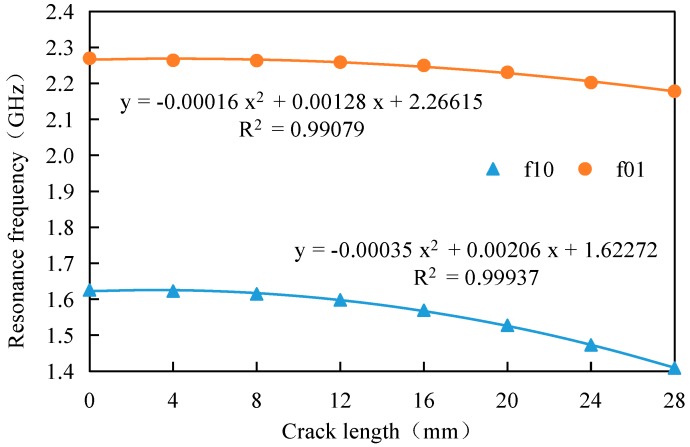
The relation between resonant frequency and crack length of oblique crack.

In order to investigate the influence of crack direction on detection sensitivity, based on the above-mentioned models, the crack angle θ is set as 30°, the crack length is also studied by a parametric study and the crack step length is set as 4 mm, as shown in Figure 8.

It can be learned from Figure 8 that the results for oblique cracks are similar to those for straight cracks. The resonant frequency decreases with increasing crack length while the sensitivity of the antenna sensor increases with increasing crack length. When the crack length is 16 mm, the sensitivity of TM_10_ and TM_01_ are 3.84 MHz/mm and 9.14 MHz/mm, respectively, which indicates that the antenna sensor designed in this paper still has a high sensitivity for oblique crack identification.

## 5. Influence of FRP Properties on Crack Monitoring

### 5.1. Influence of CFRP Conductivity on Crack Detection

In addition to GFRP, which is used in crack reinforcement on steel structures, CFRP is also frequently used in reinforcement applications. Due to the weak conductivity of orthotropic CFRP, it is necessary to analyze the effects of its conductivity on crack monitoring performance.

The numerical software that can be used to investigate antenna sensors includes High Frequency Structure Simulator (HFSS), ADS (advanced design system), and CST (computer simulation technology). However, it is not feasible to simulate the effects of a conductive substrate on the resonant frequency using numerical software. Therefore, an experimental investigation is performed to analyze the weak conductivity of CFRP in this work. To compare the results with those of previous GFRP tests, the shape and size of the antenna sensor used in this experiment were set to be the same as those used in the previous tests. The properties of the four layers in the “steel plate–CFRP–substrate–patch” configuration and the geometrical factors are shown in Table 5.

**Figure 9:**
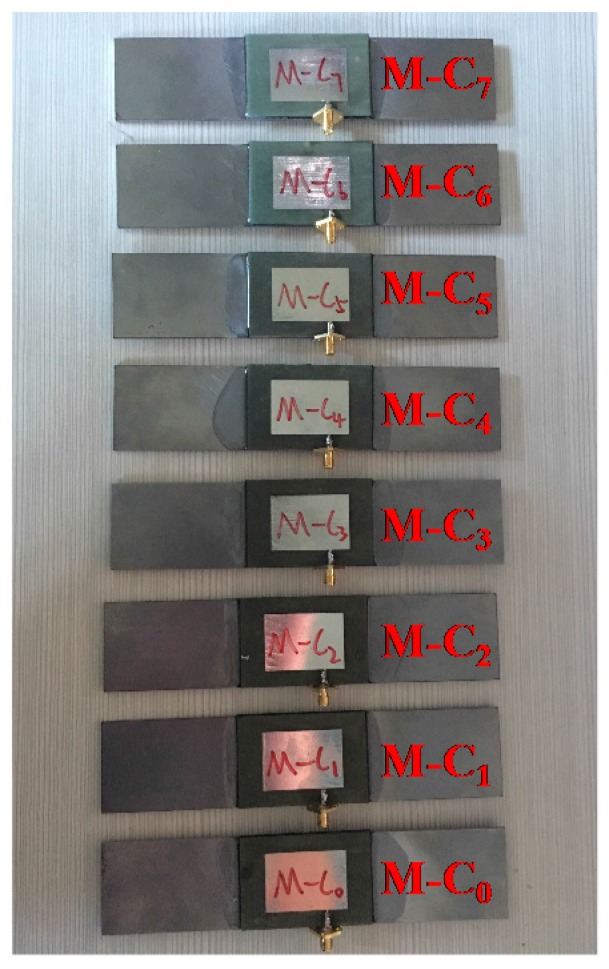
Specimens with crack under CFRP reinforcement layers.

**Figure 10:**
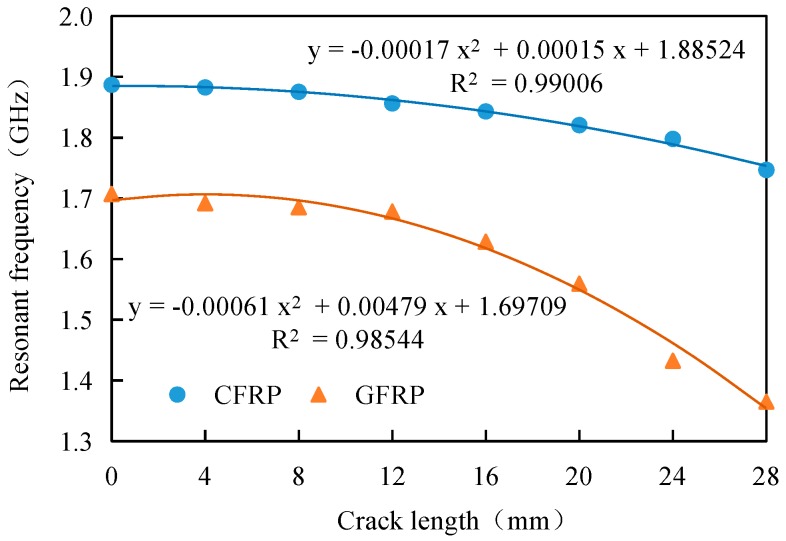
Experimental results for CFRP and GFRP.

Orthotropic CFRP was used with its conductivity set at 10,000 S/m along the direction of the two perpendicular carbon fiber layers, while the conductivity in the thickness direction was 100 S/m. The samples are shown in Figure 9, while the crack lengths that correspond to specimens M-C_0_ to M-C_7_ are 0 mm, 4 mm, 8 mm, 12 mm, 16 mm, 20 mm, 24 mm, and 28 mm, respectively. The experimental results are shown in Figure 10. A through crack with 0.5 mm width was cut by EDM (electrical discharge machining) for specimens M-C_0_ to M-C_7_ with a 0.002 mm machining accuracy.

As shown in Figure 10, a significant resonance frequency offset can still be obtained in the CFRP experiments. The experimental rules for CFRP are consistent with those of GFRP. The resonant frequency of the antenna sensor decreases with increasing crack length, and the two curves are parabolic in shape. The experimental results show that the dual-substrate antenna sensor designed in this paper is also suitable for crack monitoring in FRP-strengthened steel structures.

In Figure 10, the resonant frequency of the CFRP specimen is shown to be higher than that of the GFRP specimen for the same crack length. This is because the relative dielectric constant of CFRP is lower than that of GFRP. Based on Equations (1)–(3), this indicates that the resonant frequency decreases when the relative dielectric constant increases, which means that the fundamental resonant frequency theory of the dual-substrate antenna sensor proposed in this paper has been verified experimentally.

Figure 10 also shows that the resonant frequency offset obtained in the CFRP experiment is significantly smaller than the corresponding offset for GFRP. Due to the effects of the conductivity of CFRP, the sensitivity of the antenna sensor for crack monitoring in CFRP-strengthened steel structures is lower than that for GFRP-strengthened steel structures.

### 5.2. Influence of FRP Thickness on Crack Detection

In actual engineering applications of FRP-strengthened steel structures, the FRP thickness is a variable. In the dual-substrate antenna sensor with the four layer “steel plate–FRP–substrate–patch” configuration, the FRP thickness has a fundamental effect on the resonant frequency of the antenna sensor.

**Figure 11:**
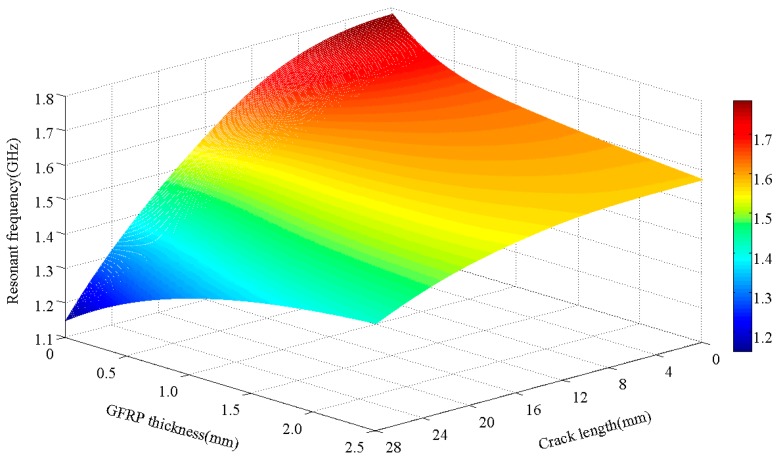
Resonant frequency–GFRP thickness–crack length characteristics in a 3D diagram.

To investigate the influence of the FRP thickness on the crack monitoring performance of the antenna sensors, the parameters of two dimensions, i.e., the GFRP thickness and the crack length, were changed simultaneously using the HFSS software. Three-dimensional surface plots of “resonant frequency–GFRP thickness–crack length” characteristics were obtained, as shown in Figure 11.

Figure 11 shows that when the crack is relatively short, the antenna sensor’s resonant frequency decreases with increasing FRP thickness; however, when the crack is relatively long, the resonance frequency of the antenna sensor then increases with increasing FRP thickness. When the crack length increases, the resonant frequency of the antenna sensor gradually decreases. The resonant frequency offset becomes smaller with increasing FRP thickness; e.g., when the crack length propagates from 0 mm to 28 mm, the resonant frequency offset is 0.63119 GHz at a crack length of 0 mm, while the resonant frequency offset is 0.16045 GHz at a crack length of 2.5 mm.

#### 5.2.1. Influence of FRP Thickness on Resonant Frequency

**Figure 12:**
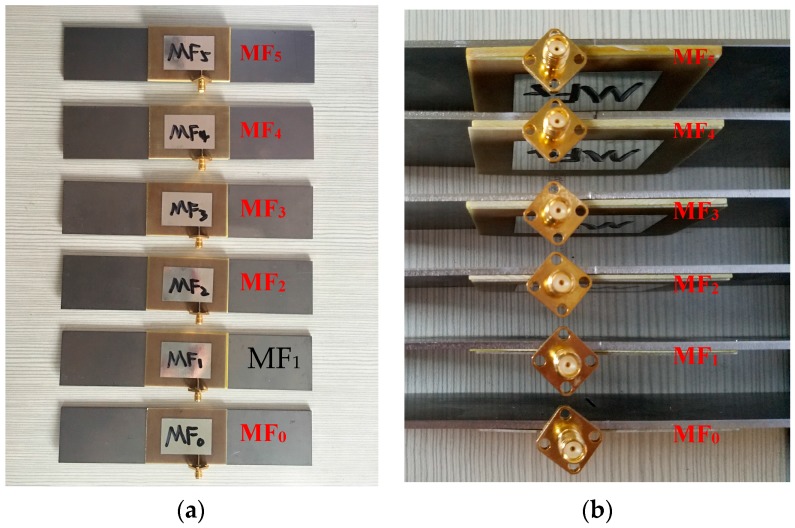
Specimens with different GFRP thicknesses. (**a**) Top view; (**b**) Side view.

**Figure 13:**
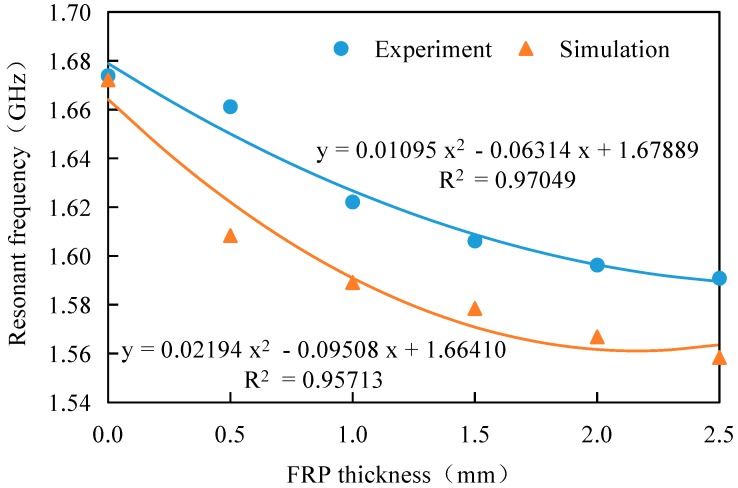
Effects of FRP thickness on resonant frequency.

A series of crack detection experiments with various FRP thicknesses were designed to investigate the effects of the FRP thickness on the resonant frequency of the antenna sensor. As shown in Figure 12, the different GFRP thicknesses corresponding to specimens MF_0_ to MF_5_ were 0 mm, 0.5 mm, 1 mm, 1.5 mm, 2 mm, and 2.5 mm, respectively. All the steel plates used in the specimens contained a crack with a length of 12 mm. After the glue solidified, the resonance frequency of each specimen was measured using a Vector Network Analyzer (VNA), as shown in Figure 13.

Figure 13 indicates that for a specimen with a 12 mm crack length, the antenna sensor resonance frequency decreases with increasing FRP thickness. The experiment results match the numerical result ideally, while the fitting coefficient of determination is higher than 0.95, meaning that the fitting results are reliable, and the resonant frequency shows a parabolic relation with the FRP thickness.

**Figure 14:**
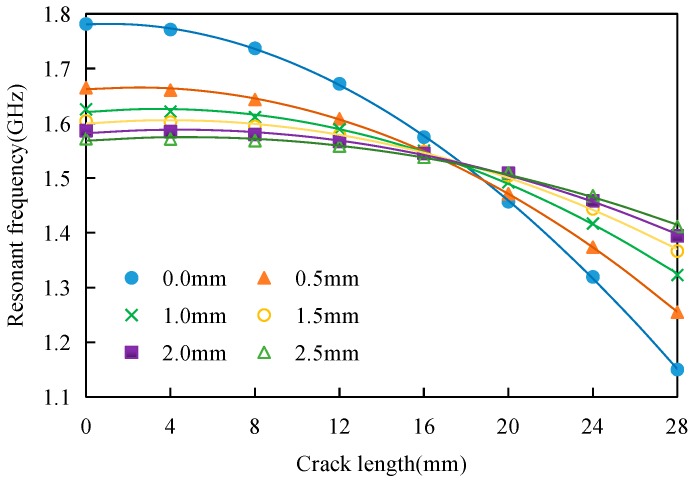
Relationship between resonant frequency and crack length for the different FRP thicknesses.

The resonant frequency–crack length relationships for the remaining crack lengths can be extracted from Figure 14. Table 6 shows the polynomial fitting results for the relationship between the resonant frequency and the crack length.

The combination of the curve in Figure 14 and the polynomial fitting equation in Table 6 shows that the resonant frequency–crack length relationship curves intersect with each other at a point with a crack length of 17 mm. This indicates that when the crack length is less than 17 mm (approximately 3/5 of the patch width), the antenna sensor’s resonant frequency then decreases with increasing FRP thickness; however, when the crack is longer than 17 mm, the resonance frequency of the antenna sensor increases, increasing FRP thickness.

#### 5.2.2. Influence of FRP Thickness on Sensitivity

Figure 14 indicated that the resonant frequencies of the antenna sensors with different FRP thicknesses all decrease with increasing crack length. A thicker FRP layer leads to a smoother resonant frequency–crack length curve, which indicates that the sensitivity of the antenna sensor decreases with increasing FRP thickness.

**Figure 15:**
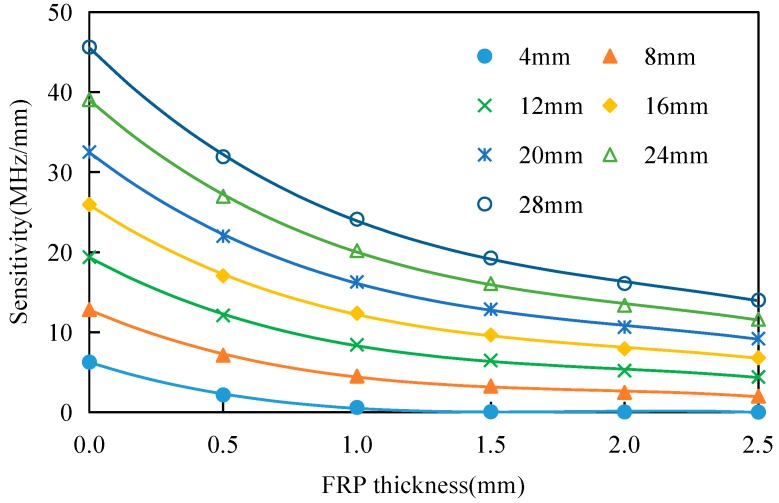
Effects of Fiber Reinforced Polymer (FRP) thickness on the antenna sensor’s sensitivity.

In Table 6, via polynomial fitting of the resonant frequency–crack length relationship for the different FRP thicknesses, the coefficients of determination are all higher than 0.99, which indicates that these fitting results are reliable. Based on the polynomial equation that was fitted in Table 6, the sensitivity of the proposed antenna sensor for the monitoring of cracks of different lengths under different FRP thicknesses can be obtained, which are shown in Figure 15.

Figure 15 shows that the sensitivity of the antenna sensor decreases gradually with increasing FRP thickness. For a crack with a length of 8 mm, the sensitivity of the antenna sensor is 7.13 MHz/mm when the FRP thickness is 0.5 mm, but when the FRP thickness increases to 2.5 mm, the sensitivity is reduced to 2.01 MHz/mm. For a crack with a length of 20 mm, the sensitivity of the antenna sensor is 22.01 MHz/mm when the FRP thickness is 0.5 mm, but when the FRP thickness increases to 2.5 mm, the sensitivity is then reduced to 9.21 MHz/mm.

## 6. Effects of Strain on Crack Monitoring

Under actual working conditions, FRP-strengthened steel structures may be affected by strain and cracking simultaneously. The results of Yi et al. [25] and Wang et al. [21] show that the strain can lead to a change in the size of the patch so that the resonance frequency of the antenna sensor is then shifted. Therefore, a study of the influence of strain on antenna sensors is highly valuable for accurate crack identification.

**Figure 16:**
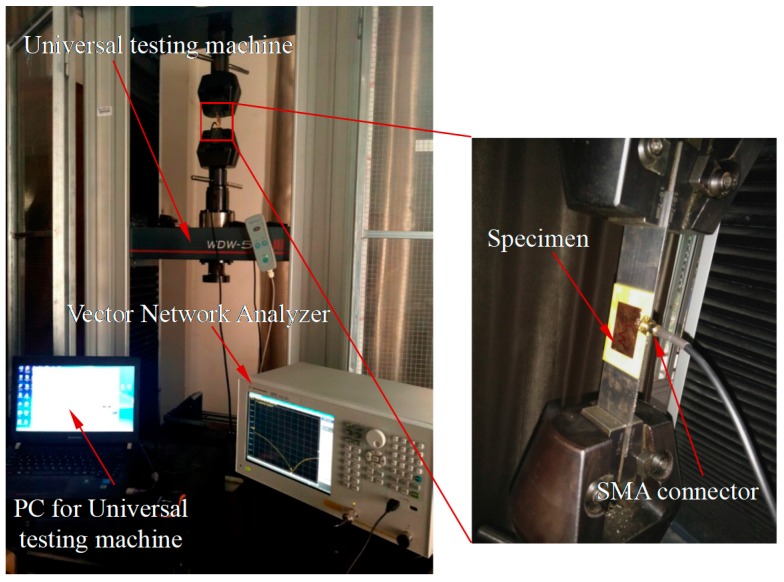
Experimental platform used for strain detection of antenna sensor.

To discuss this issue, a strain measurement experiment was designed for the antenna sensor. The antenna sensors M_1_ to M_7_ were placed on the WDW-50E microcomputer controlled electronic universal testing machine to apply tension, as shown in Figure 16.

The universal testing machine was programmed to apply loads ranging from 0 με to 1000 με with increments of 200 με based on the cross-sectional areas of the specimens. When the tensile load is 18.480 kN, the corresponding microstrain and stress are 1000 and 210 MPa, respectively. This load is appropriate for the majority of Q235 steel working conditions. At each load increment step, there was a 60 s pause to measure the S_11_ curve of the antenna sensor. The results of these strain experiments are summarized in Table 7.

Table 7 indicates that the antenna sensor’s resonance frequency decreases with increasing strain. When the microstrain of the steel plate is 1000, the variation among the resonant frequencies of M_1_, M_2_ and M_3_ is only approximately 2 MHz. Based on the resonant frequency–crack length fitting relationship, when the crack length is 9.3 mm (which is approximately 1/3 of the patch width), a resonant frequency variation of 2 MHz corresponds to an increment in the crack length of 0.3 mm. However, when the crack length is 18.7 mm (or approximately 2/3 of the patch width), the resonant frequency variation of 2 MHz corresponds to an increment in the crack length of 0.1 mm. This indicates that the strain has only a tiny influence on the crack monitoring performance of the antenna sensors, and it can therefore be neglected during the crack monitoring process.

## 7. Conclusions

In this paper, an antenna-sensor-based method for the monitoring of crack damage in FRP-strengthened steel structures has been studied, and the conclusions are summarized as follows.

1. The antenna sensor can detect cracks in an FRP-strengthened steel structure. The resonant frequency decreases with increasing crack length, while the sensitivity of the antenna sensor increases with increasing crack length.
2. The weak conductivity of CFRP interferes with the test signal, but the effective resonance frequency offset can still be measured. The dual-substrate antenna sensor designed here is also suitable for use with CFRP-reinforced steel structures.
3. The FRP layer thickness has a significant influence on the antenna sensor’s resonant frequency. When the crack length is less than 17 mm (approximately 3/5 of the patch width), the sensor antenna resonant frequency decreases with increasing FRP thickness. However, when the crack length is more than 17 mm, the antenna sensor’s resonant frequency then increases with increasing FRP thickness.
4. The sensitivity of the antenna sensor used to monitor the cracking of FRP-strengthened steel structure decreases with increasing FRP thickness.
5. Strain has very little effect on the antenna sensor’s ability to monitor cracking under the FRP coverage.
6. The minimum crack size detected by an antenna sensor designed in this paper is less than 0.5 mm.
7. In order to improve the sensitivity and robustness of the antenna sensor, it is necessary to strictly control the welding process or adopt wireless testing to avoid the instability caused by the SMA joint. The manufacturing accuracy of patch antenna sensors is also an aspect that needs to be improved.

## 8. Future Prospects

1. High frequency antenna sensors will be more sensitive to crack identification, and we will design higher frequency antenna sensors in future work.
2. The identification of multiple cracks by a single antenna sensor involves complex current distribution, which can be studied from the influence of multiple cracks on the antenna current path.

## Figures and Tables

**Table 1:** Geometrical dimensions and material properties of dual-substrate antenna sensor layers.

Material	Length (mm)	Width (mm)	Thickness (mm)	The Dielectric Constant
Patch	40	28	0.05	/
Substrate	64	44	0.4	4.4
GFRP	64	44	1	5.95
Steel plate	200	44	2	/

**Table 2:** Crack detection sensitivity of antenna sensor.

Crack Length (mm)	Simulation (MHz/mm)	Experiment (MHz/mm)	Difference (%)
4	0.09	0.6	/
8	4.97	4.52	9.05
12	9.85	8.44	14.31
16	14.73	12.36	16.09
20	19.61	16.28	16.98
24	24.49	20.2	17.52
28	29.37	24.12	17.88

**Table 3:** The sensitivity of small scale crack size.

Crack Length (mm)	Sensitivity (MHz/mm)
0.5	0.1
1	0.31
1.5	0.52
2	0.73
2.5	0.94
3	1.15
3.5	1.36
4	1.57

**Table 4:** Detection results of crack direction.

Crack Orientation (°)	*f*_10_ (GHz)	*f*_01_ (GHz)	*R*
0	1.62154	2.26665	1.3978
15	1.62192	2.26632	1.3973
30	1.62223	2.26596	1.3968
45	1.62279	2.26515	1.3958
60	1.6232	2.26467	1.3952
75	1.62348	2.26404	1.3946
90	1.62411	2.26315	1.3935

**Table 5:** Geometrical factors and material properties of carbon fiber reinforced polymer (CFRP).

Material	Length (mm)	Width (mm)	Thickness (mm)	The Dielectric Constant
Patch	40	28	0.05	/
Substrate	64	44	0.4	4.4
CFRP	64	44	1	4.17
Steel plate	200	44	2	/

**Table 6:** Polynomial fitting results for the relationship between resonant frequency and crack length.

FRP Thickness (mm)	Polynomial Fitting Equation	Decision Coefficient *R*^2^
0.0	*y* = −0.00082*x*^2^ + 0.00029*x* + 1.78335	0.99982
0.5	*y* = −0.00062*x*^2^ + 0.00279*x* + 1.66248	0.99983
1.0	*y* = −0.00049*x*^2^ + 0.00332*x* + 1.62070	0.99896
1.5	*y* = −0.00040*x*^2^ + 0.00313*x* + 1.59963	0.99832
2.0	*y* = −0.00034*x*^2^ + 0.00295*x* + 1.58196	0.99712
2.5	*y* = −0.00030*x*^2^ + 0.00279*x* + 1.56812	0.99723

**Table 7:** Influence of strain on crack detection performance of antenna sensor.

με	Load (kN)	M_1_ (GHz)	M_2_ (GHz)	M_3_ (GHz)	M_4_ (GH)z	M_5_ (GHz)	M_6_ (GHz)	M_7_ (GHz)
0	0	1.69219	1.68544	1.67825	1.62853	1.5595	1.43265	1.36525
200	3.696	1.69175	1.68519	1.67813	1.6283	1.55925	1.43235	1.3651
400	7.392	1.69156	1.68425	1.67806	1.62815	1.55906	1.43195	1.36405
600	11.088	1.69138	1.68406	1.67781	1.62778	1.5585	1.43085	*
800	14.784	1.69106	1.68375	1.67763	1.6253	1.55644	*	*
1000	18.48	1.69094	1.68331	1.67644	1.62245	1.55113	*	*

* The cross-sectional area where the crack was located in specimens M_6_ and M_7_ was less than 1/2 of the original cross-sectional area of the steel plate. For safety reasons, the resonant frequencies of the antenna sensor were measured under partial strain.

## References

[1] Eid R., Paultre P. (2017). Compressive Behavior of FRP-Confined Reinforced Concrete Columns. Eng. Struct..

[2] Heshmati M., Haghani R., Al-Emrani M. (2016). Effects of moisture on the long-term performance of adhesively bonded FRP/steel joints used in bridges. Compos. Part B Eng..

[3] Luo M., Li W., Hei C., Song G. (2016). Concrete Infill Monitoring in Concrete-Filled FRP Tubes Using a PZT-Based Ultrasonic Time-of-Flight Method. Sensors.

[4] Tang Y., Wu Z. (2016). Distributed Long-Gauge Optical Fiber Sensors Based Self-Sensing FRP Bar for Concrete Structure. Sensors.

[5] Jiang M., Sai Y., Geng X., Sui Q., Liu X., Jia L. (2016). Development of an FBG Sensor Array for Multi-Impact Source Localization on CFRP Structures. Sensors.

[6] Colombi P., Fava G., Sonzogni L. (2015). Fatigue crack growth in CFRP-strengthened steel plates. Compos. Part B Eng..

[7] Naidjate M., Helifa B., Feliachi M., Lefkaier I.K., Heuer H., Schulze M. (2017). A Smart Eddy Current Sensor Dedicated to the Nondestructive Evaluation of Carbon Fibers Reinforced Polymers. Sensors.

[8] Colombi P., Fava G. (2016). Fatigue crack growth in steel beams strengthened by CFRP strips. Theor. Appl. Fract. Mech..

[9] Yu Q., Chen T., Gu X., Zhao X. (2016). Boundary element analysis of edge cracked steel plates strengthened by CFRP laminates. Thin-Walled Struct..

[10] Li X., Liu Z., Jiang X., Lodewijks G. (2016). Method for detecting damage in carbon-fibre reinforced plastic-steel structures based on eddy current pulsed thermography. Nondestruct. Test. Eval..

[11] Wang Y. (2016). Monitoring of Crack Propagation in Fibre-Reinforced-Polymer (FRP) Strengthened Steel Plates Using Guided Waves. Master′s Thesis.

[12] Ma G., Li H. (2017). Acoustic emission monitoring and damage assessment of FRP-strengthened reinforced concrete columns under cyclic loading. Constr. Build. Mater..

[13] Deshmukh S., Mohammad I., Tentzeris M., Wu T., Huang H. (2009). Crack Detection and Monitoring Using Passive Wireless Sensor. ASME Conf. Smart Mater..

[14] Mohammad I., Huang H. (2010). Monitoring fatigue crack growth and opening using antenna sensors. Smart Mater. Struct..

[15] Mohammad I., Gowda V., Zhai H., Huang H. (2012). Detecting crack orientation using antenna sensors. Meas. Sci. Technol..

[16] Cook B.S., Shamim A., Tentzeris M.M. (2012). Passive low-cost inkjet-printed smart skin sensor for structural health monitoring. IET Microw. Antennas Propag..

[17] Yi X., Cho C., Wang Y., Tentzeris M.M. (2016). Battery-free slotted patch antenna sensor for wireless strain and crack monitoring. Smart Struct. Syst..

[18] Liu M., Li B., Li H. (2015). A crack monitoring method based on microstrip patch antenna. Proc. Annu. Reliab. Maintainab. Symp..

[19] Yi X., Wang Y. Thickness variation study of RFID-based folded patch antennas for strain sensing. Proceedings of the SPIE Smart Structures and Materials & Nondestructive Evaluation and Health Monitoring.

[20] Yi X., Vyas R., Cho C., Fang C.H., Cooper J., Wang Y., Leon T.R., Tentzeris M.M. (2012). Thermal effects on a passive wireless antenna sensor for strain and crack sensing. Proc. SPIE.

[21] Wang W.J., Ren X., Sun Y., Li H., Liu M.B. (2016). Development of a strain measurement method utilizing a rectangular microstrip patch antenna. Int. J. Appl. Electromagn. Mech..

[22] Biswas M., Sen M. (2014). Design and Development of Rectangular Patch Antenna with Superstrates for the Application in Portable Wireless Equipments and Aircraft Radome. Microw. Opt. Technol. Lett..

[23] Wheeler H.A. (1964). Transmission-Line Properties of Parallel Wide Strips by a Conformal-Mapping Approximation. IEEE Trans. Microw. Theory Tech..

[24] Mohammad I., Huang H. (2015). An Antenna Sensor for Crack Detection and Monitoring. Adv. Struct. Eng..

[25] Yi X., Cho C., Cooper J., Wang Y., Tentzeris M.M., Leon R.T. (2013). Passive wireless antenna sensor for strain and crack sensing-electromagnetic modeling, simulation, and testing. Smart Mater. Struct..

